# Involvement of neuronal factors in tumor angiogenesis and the shaping of the cancer microenvironment

**DOI:** 10.3389/fimmu.2024.1284629

**Published:** 2024-02-05

**Authors:** Sharif Shalabi, Ali Belayachi, Bruno Larrivée

**Affiliations:** ^1^ Maisonneuve-Rosemont Hospital Research Center, Boulevard de l’Assomption, Montréal, QC, Canada; ^2^ Department of Biochemistry and Molecular Medicine, Montréal, QC, Canada; ^3^ Ophthalmology, Université de Montréal, boul. Édouard-Montpetit, Montréal, QC, Canada

**Keywords:** angiogenesis, neurovascular crosstalk, growth factors (angiogenesis factors), tumor microenvironment, tumorigenesis

## Abstract

Emerging evidence suggests that nerves within the tumor microenvironment play a crucial role in regulating angiogenesis. Neurotransmitters and neuropeptides released by nerves can interact with nearby blood vessels and tumor cells, influencing their behavior and modulating the angiogenic response. Moreover, nerve-derived signals may activate signaling pathways that enhance the production of pro-angiogenic factors within the tumor microenvironment, further supporting blood vessel growth around tumors. The intricate network of communication between neural constituents and the vascular system accentuates the potential of therapeutically targeting neural-mediated pathways as an innovative strategy to modulate tumor angiogenesis and, consequently, neoplastic proliferation. Hereby, we review studies that evaluate the precise molecular interplay and the potential clinical ramifications of manipulating neural elements for the purpose of anti-angiogenic therapeutics within the scope of cancer treatment.

## 1 Introduction

The tumor microenvironment plays a critical role in the progression of solid malignancies and will alter the response of cancers to therapeutic approaches such as chemotherapy, radiotherapy, or immunotherapy (1–4). The tumor microenvironment encompasses a complex network of multiple cell types, including fibroblasts, vascular endothelial cells, pericytes, adipocytes, neurons, stem cells, and immune cells (5). Interactions between tumor, stromal cells as well as the extracellular matrix alter the phenotypic properties of tumor cells that can facilitate their dissemination. As such, a better understanding of the underlying cellular and molecular mechanisms governing these interactions could lead to the development of novel therapeutic strategies to fight cancer.

Endothelial cells, as part of the tumor vasculature, play an integral role in defining the properties of the tumor microenvironment. Tumor blood vessels, established primarily through angiogenesis, are required to bring oxygen and nutrients to the tumors, as well as to facilitate tumor cell dissemination. However, the tumor vasculature is often highly dysfunctional and poorly perfused, leading to hypoxia, significantly reshaping the tumor microenvironment (6). As such, vascular normalization, which aims to restore the functionality of the vasculature in solid cancers and restore perfusion, could represent an important strategy to modulate the microenvironment within tumors. A better understanding of the defects associated with pathological tumor angiogenesis would be key to identify novel targets to facilitate vascular normalization.

During development, blood vessels and peripheral nerves form highly branched networks that interact and regulate each other’s patterning (7). Indeed, alignment of nerves and blood vessels allows the establishment of a physical relationship between these two systems, and they also respond to related families of guidance cues and growth factors during the establishment of their respective patternings (8). In addition to tumor angiogenesis, recent evidence points to the ability of tumors to recruit neurons (neoneurogenesis) and stimulate innervation (axonogenesis) (9). The presence of neuronal cells has been shown to impact the tumor microenvironment through their interactions with tumor cells, immune cells, and endothelial cells. Furthermore, cancer cells can also infiltrate nerves, a process referred to as perineural invasion (10), demonstrating that nerve fibers in tumors can also act as a mechanical support that enables and facilitates cancer cell migration. Despite the close association of nerves and blood vessels during development, the contribution of neurons to tumor angiogenesis remains unclear, although several studies show that neurons within the tumor microenvironment modulate vascular function in tumors (11). This review summarizes the current state of knowledge regarding the interactions between blood vessels and nerves in homeostasis and cancer, including 1) physiological neurogenesis and angiogenesis and evidence of their interdependence, 2) neurogenesis and angiogenesis in the context of cancer and how they are regulated by one another and 3) the function of neurotransmitters/neuropeptides, axon guidance molecules and neurotrophic factors in neuro-tumoral cooperation, and the distinct involvement of sympathetic and sensory nerves in cancer progression.

## 2 Physiological and pathological angiogenesis

### 2.1 Developmental angiogenesis

Angiogenesis, the growth of blood vessels from the existing vasculature, occurs in both physiological and pathological contexts to ensure the transport of oxygen, nutrients, and metabolic waste to tissues. The inner wall of blood vessels is made of endothelial cells that form a monolayer, interconnected by junctional molecules such as VE-cadherin and claudins (12). Endothelial cells interact with a basal membrane and are surrounded by mural cells that prevent vascular leakage by limiting hyperpermeability. The contractility of mural cells further contributes to vessel diameter and the regulation of blood pressure (13).

Embryonic vascular development commences with the formation and aggregation of angioblast precursors within the embryo proper, leading to the creation of blood islands in the visceral yolk sac (14). Subsequently, vasculogenesis occurs, in which blood islands come together to establish a primitive network of endothelial tubes, followed by angiogenesis, which orchestrates the expansion and maturation of vessels through intricate molecular mechanisms, encompassing endothelial cell proliferation and remodeling of the initial vascular network (15). Ultimately, the differentiation of these primitive blood vessels into arteries or veins relies on both intrinsic and extrinsic factors. While vessels may exhibit a genetic predisposition for specific phenotypes, external influences such as haemodynamic forces, which encompass shear stress and blood pressure, can also impact the outcome of blood vessel differentiation (16).

During angiogenic sprouting, Vascular Endothelial Growth Factor (VEGF) stimulates endothelial cells, causing them to become pro-migratory and to adopt a tip cell phenotype (17). Their filopodia and motility increase their responsiveness to the extracellular matrix, growth factors and attractive or repulsive signaling molecules to guide their patterning. Therefore, activated tip cells initiate sprouting whereas following endothelial cells, referred to as stalk cells, proliferate to establish the structural and functional integrity of nascent vessels (17). Several bodies of evidence have shown that the nervous system, through the release of growth factor and axon guidance cues, participates in tip cell guidance and stalk cell elongation during the angiogenic process. For example, it has been shown that several axon-regulating molecules belonging to the Slit/Robo, netrin/DCC/Unc5 and neuropilin/plexin/sema families mediate proper vascular patterning (18).

### 2.2 Tumor angiogenesis

Tumor blood vessels are dilated, disorganized and immature which, combined with a lack of mural cells association, leads to excessive permeability, poor perfusion and increased hypoxia (19). The important secretion of VEGF by tumor cells 1) suppresses PDGFRβ (platelet-derived growth factor receptor beta) signaling in vascular smooth muscle cells via the complexation of PDGFRβ and VEGFR2 that assemble into a hybrid receptor, and 2) modulates the activity of GTPases like RhoA with the effect of increasing vascular permeability (20, 21). The junctional integrity of the vessels is compromised following the release of proteolytic enzymes by tumor cells such as Matrix Metalloproteinases (MMPs), elastase and trypsin that that can accelerate VE-cadherin cleavage together with VEGF signaling (22). The release of inflammatory factors such as histamine further contributes to vessel leakages by increasing the permeability of endothelial cells (23). Given that cancer is characterized by uncontrolled cell growth, the surrounding host tissue restricts tumor expansion which induces mechanical forces of compression that collapse intratumoral vessels, thus suppressing blood flow and lymphatic network functionality by hindering the drainage of excessive interstitial fluid (24). Blood vessel compression limits perfusion, thereby decreasing oxygen and nutrient concentrations, resulting in a hypoxic microenvironment that selects aggressive clones that activate oncogenes and undergo an epithelial-to-mesenchymal transition, which amplifies the metastatic potential of cancer cells (25). Furthermore, reduced perfusion within tumors is well known to limit the therapeutic efficacy of chemotherapy and radiotherapy treatments. Hypoxia is a major driver of tumor angiogenesis. Under normoxic conditions, prolyl hydroxylase domain protein 2 (PHD2) catalyzes the hydrolysis of hypoxia-inducible factors (HIF) transcription factors with oxygen as the co-factor, which target them for ubiquitination by the Von Hippel-Lindau complex and subsequent degradation by the proteasome (26, 27). Under hypoxic conditions, PHD2 becomes inactive, resulting in HIF1 escaping degradation and binding to hypoxia response elements, a DNA motif present in HIF target genes. Increased levels of HIF proteins lead to the expression of genes involved in cellular adaptation against acute and chronic hypoxia such as angiogenesis (28).

## 3 The nervous system and angiogenesis

It is well-documented that a strong interplay exists between the neuronal and vascular systems. Individually, nerves and blood vessels maintain normal body function, however, the intricate parallel of anatomic and functional growth between the two has also been deemed necessary for enabling normal function, development, and homeostasis of tissues.

At the anatomical level, arteries and nerves often course alongside each other, exhibiting a high degree of organizational similarity (8).

### 3.1 Neural activity effects on angiogenesis

There is a strong interdependence between blood vessels and neurons, as neurons have a high metabolic demand, which require blood vessels supplying them with nutrients and oxygen and protecting them from pathogens and toxins. In turn, nerves control blood vessel diameter, heart rate, and other hemodynamic attributes thus linking their functional independence with their proximity.

The modality of the strong interplay between nerves and blood vessels varies slightly between the central nervous system (CNS) and the peripheral nervous system (PNS). In the CNS, multiple different neuronal cell types play a role in the differentiation of blood vessels with a conventional pattern of large brain arteries. While in the PNS, the nerves and blood vessels run in parallel to each other due to a phenomenon named “neurovascular congruency” (29).

Studies have documented the crucial link between neural activity and the vascularization of the nervous system. For example, in the visual cortex, inhibition or promotion of neural activity by modulating visual stimuli significantly modulated vascular density (30, 31). Experiments in the mouse primary somatosensory cortex have also demonstrated that somatosensory stimuli are critical in shaping cortical blood vessels (32). These data support the hypothesis that neural activity driven by sensory experiences can shape the development of the vasculature. Accordingly, reduced glutamatergic neurotransmitter release at the thalamocortical synapses in transgenic mice, leading to diminished neuronal activity, also led to decreased vessel density and branch points, suggesting that altered activity along the somatosensory axis results in changes in cortical angiogenesis (33).

Several neurotransmitters have been shown to play a significant role during physiological angiogenesis. Dopamine release has been shown to significantly delay angiogenesis during wound healing, and D2 dopamine receptor antagonists significantly accelerated wound healing in mice by inducing angiogenesis, in part through its up-regulation of α5β1 integrin (34). Dopamine was also shown to inhibit vascular development in the retina by restricting VEGFR activation and Notch-Jagged1 signaling (35). GABA and its receptor signaling have also been shown to shape neurovascular interactions and angiogenesis during embryonic brain development. Endothelial cell-specific conditional mouse models of the GABA pathway (Gabrb3ΔTie2-Cre) resulted in a significant reduction in the vascular density of the embryonic brain which persisted in the adult brain, accompanied by concurrent GABAergic neuronal cell deficits (36). Mice lacking serotonin in the nervous system (*Tph2-/-*), while not showing obvious angiogenic defects, also displayed altered cardiovascular parameters, including decreased blood pressure, due to diminished sympathetic nerve activity (37). Nicotine, an agonist of the acetylcholine receptor (AChR), was shown to enhance physiological angiogenesis, and improve wound healing or limb ischemia (38, 39). The nicotinic acetylcholine receptor α3 subtype (α3-nAChR) was also shown to modulate vascular inflammation by restricting inflammatory cells infiltration and the release of inflammatory cytokines (40). Finally, metabotropic glutamate receptors (mGluR) were also shown to play important roles in the maintenance of vascular integrity. Treatment of human brain endothelial cells with glutamate or selective mGluR group I or III agonists resulted in a time-dependent loss of phosphorylated vasodilator-stimulated phosphoprotein (VASP) and significantly increased endothelial permeability. *In vivo*, mGluR antagonists significantly decreased blood-brain barrier function (41). Altogether, these studies highlight the multifaceted roles of neurotransmitters in vascular development and function.

### 3.2 Axon guidance molecules and angiogenesis

**Figure 1:**
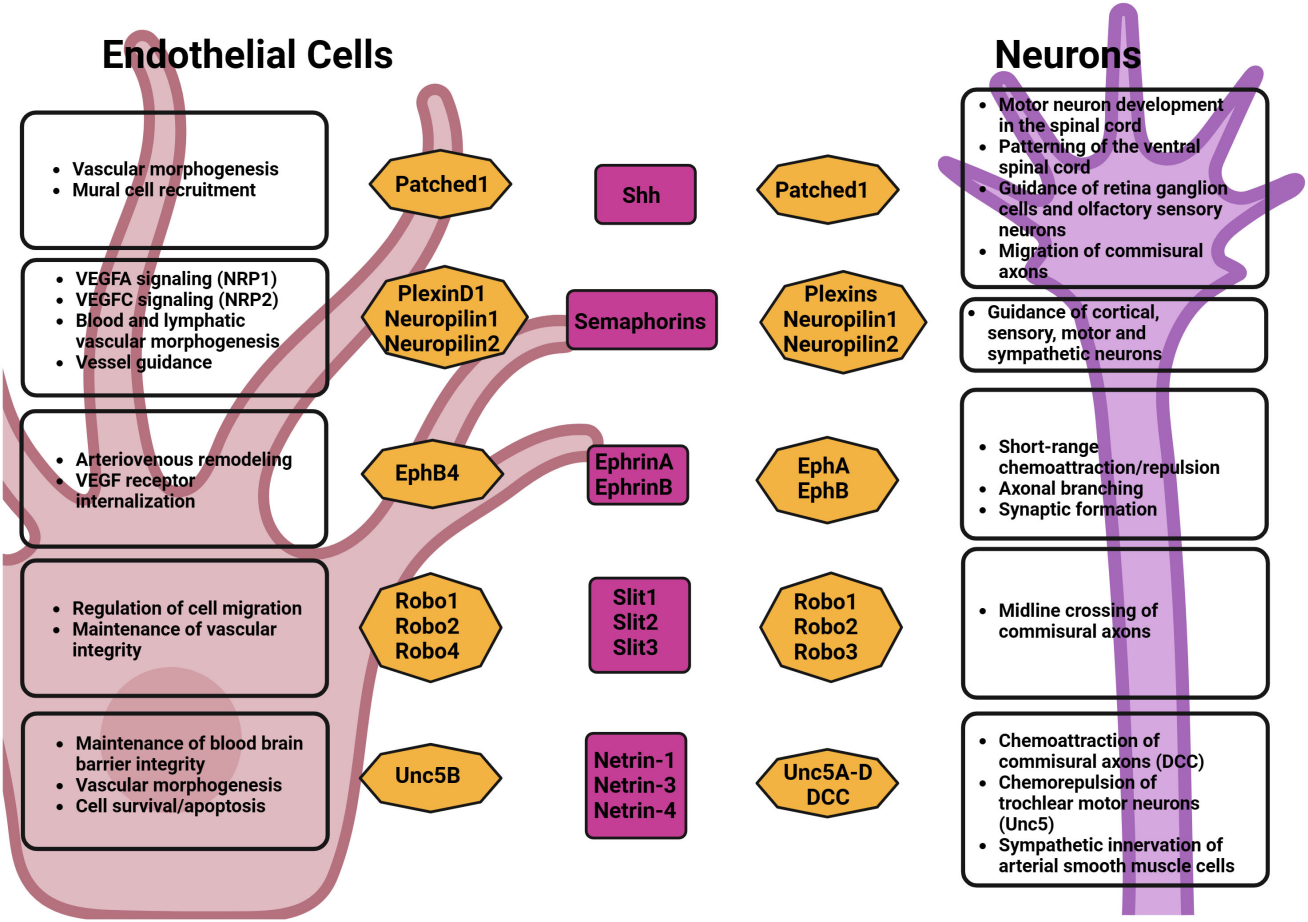
Axonal growth cues effects on neurons and endothelial cells.

At the cellular level, endothelial tip cells and axonal growth cones display striking similarities in both their morphological structures and functions: endothelial tip cells play a crucial role in guiding the development of blood vessels, while growth cones are found at the leading edges of growing axons (42). At the molecular level, endothelial tip cells and axonal growth cones have also been shown to respond to similar families of growth factors and comparable guidance cues (**Figure 1**) (18).

For example, VEGF, aside from its role in vascular development, has also been shown to participate in the development of the CNS by promoting axonal and dendrite growth *in vivo* and *in vitro (* 43–45). In the PNS, VEGF was shown to induce axonal growth in dorsal root ganglia by attracting growth cone (46) and was also shown to promote Schwann cell proliferation and migration through VEGFR2 (47). Several other neural guidance factors and receptors, which were first identified in the patterning of the nervous system, were also found to play a role in the establishment of the vasculature. These include the Semaphorins, Slits, Eph family, and Netrins (18).

*Semaphorins/plexins/neuropilins:* Semaphorins were initially identified as axonal cone guidance molecules. They have however since been described to have numerous functions in various organs in the body with a variety of different functions. Semaphorins are ligand molecules that may be subclassified into different groups (from classes 1-8) (48). Several of the Semaphorins’ effects, in particular their effects on the nervous system and blood vessel formation are mediated by their receptors, the Plexins and Neuropilins (49). Plexins, which were first identified on the surface of axon growth cones of dorsal root ganglia (DRG), cortical, sensory, motor and sympathetic neurons and play a role in signal transduction to steer axon growth away from the source of semaphorin, were also shown to be involved in several processes such as angiogenesis, immunoregulation, and neuronal connectivity (50).

The Semaphorins-Plexin complex plays a crucial role in blood vessel pathfinding. The main classes of semaphorins that have been shown to modulate the patterning of neurons as well as lymphatic and blood vessels are Sema3A, Sema3E, and Sema6D (51–57). Although they share a similar mechanism of action, the effects of these ligands on blood vessels and neuronal development vary substantially on account of their pattern of expression.

Loss of Plexin D1 function was shown to cause dramatic mispatterning of developing intersegmental vessels in zebrafish, which are no longer restricted to growth near intersomitic boundaries. Conversely, semaphorin overexpression inhibits the growth of intersegmental vessels in a Plexin D1-dependent manner (58). Semaphorins also interact with neuropilins, transmembrane glycoproteins that regulate neurogenesis and angiogenesis by complexing with Plexin receptors/class-3 semaphorin ligands and VEGF receptors. Neuropilin-1 and Neuropilin-2 have been shown to play crucial roles in blood and lymphatic vascular development by regulating VEGF-A and VEGF-C signaling respectively (59–61).

Sema3A, which acts to halt the extension of the axons by collapsing the growing tip or growth cones of axons, has been shown to have a role in the organization of blood vessels alongside peripheral neuronal growth *in vitro*. The fetal skin of Sema3A-deficient mice shows a disorganized peripheral vasculature running parallel to a disorganized PNS, further indicating that the peripheral nerve branching may serve as a template for the development of the developing arterial system (62).

Sema3E is highly expressed in the somites during development, where it acts as a repulsive cue for plexin-D1–expressing endothelial cells of adjacent intersomitic vessels (63). Studies have also demonstrated Sema3E’s regulatory effect on VEGF-induced Dll4-Notch signaling. A study conducted on a model of the retinal vasculature of mice concluded that VEGF directly controlled the expression of Plexin-D1, and in turn, the VEGF-induced Delta-like 4-notch signaling pathway was negatively inhibited by the Sema3E-Plexin-D1 complex. This study also found that although Sema3E remains relatively constant throughout the environment of the retina, the expression of Plexin-D1 is controlled by VEGF both temporally and spatially and that there is a role of Sema3E–Plexin-D1 function in modulating angiogenesis via a VEGF-induced feedback mechanism (55).

A knockout model of Sema6D in zebrafish demonstrated a disrupted pattern of intersegmental vessels and primary motor neurons indicating a necessary role of Sema6D in neuronal and endothelial guidance (64). Furthermore, a 2016 study using samples of human gastric cancer tissues has also shown an increased prevalence of Sema6D and Plexin A1 in endothelial vascular cells and an association of Sema6D and plexin-A1 with VEGFR2. The study provided evidence of an *in-vitro* role in angiogenesis and an increased possibility of tumor angiogenesis by VEGFR2 regulation (56). A study published by Toyofuku et al. showed a proangiogenic potential of Sema6D in conjunction with its receptor, Plexin-A1, due to its ability to aid in ventricular endocardial cell migration via the aid of VEGFR2 (65).

*Ephrin/Eph*: Ephrin ligands are regulators of neuronal and vascular development, which are classified into two groups, Ephrin As and Bs (66). Ephrins and their corresponding receptors, called Eph receptors, are involved in cell communication and guidance through bidirectional signaling (67). The role of ephrins in angiogenesis is complex and involves both stimulatory and inhibitory effects, depending on the context and specific types of ephrins and Eph receptors involved. The ephrins bind to to the ephs tyrosine kinase receptors, which can be subclassified into two classes: EphA (EphA1-EphA8) and EphB (EphB1-Eph4 & EphB6). In the nervous system, ephrins can act as short-range chemoattractants and repellents, and are important in regulating the branching of axons, dendritic morphology, and synapse formation in the developing CNS (68, 69).

In the vascular system, EphrinB2 and EphB4 are expressed in arterial and venous endothelial cells respectively, where they play a crucial role in arteriovenous specification. Targeted inactivation of the genes encoding ephrin-B2 and EphB4 in mice has shown that both are crucial for angiogenic remodeling and embryonic survival (70, 71). Ephrins are involved in the application of hemodynamic factors to the growth of the lumen as well as for the recruitment of mural cells (72). It is also known that EphrinB2 is implicated in VEGFR3 and VEGFR2 internalization processes and therefore participates in the regulation of lymphangiogenesis and angiogenesis (73).

*ROBO/Slit:* Slits and their receptors, Robo, were initially recognized as factors that attract and repel neurons across the midline aiding in neuronal axon pathfinding, branching, and migration. Indeed, it was demonstrated that specific combinations of Robo receptors in developing axons of the vertebrate spinal cord determine the final destination of these axons after crossing the midline (74). Slits and Robos have also been found to have significant roles in the vascular system. Slit2 and Slit3 are secreted by vascular smooth muscle cells, endothelial cells, and perivascular cells while Robo4, Robo1 and Robo2 have been reported to be expressed in endothelial cells (75).

It was shown that Slit2, acting through Robo1 and Robo2, promotes the migration of endothelial cells by regulating VEGF-induced Rac1 activation and lamellipodia formation (76). Robo4, an endothelial-specific isoform of the Robo family, has also been shown to promote vessel stabilization and prevent permeability through its interaction with Unc5B (77). Other studies have also shown that it can stimulate filopodia formation, cell migration, and angiogenesis suggesting that its biological effects may be context-dependent (78). It was found that the effects of Slit/Robo signaling on angiogenesis were tissue and ligand-dependent. Using Slit2/Slit3 KO mice, it was observed that stromal rather than epithelial Slit inhibit vascular growth by downregulating VEGF via Robo4, and the loss of Slit in the stroma led to an increase in blood vessel vascularity and blood vessel complexity in the mammary gland (79).

*SHH:* Sonic hedgehog (Shh), which is involved in embryonic morphogenesis, plays a crucial role in the developing nervous system, with multiple functions including the regulation of motor neuron development in the spinal cord, patterning of the ventral spinal cord, guidance of retinal ganglion cell neurons and olfactory sensory neurons, as well as the migration of commissural axons (80). Shh is secreted as a monomer, multimer, or in exovesicles, and exerts its cellular function through its interaction with the transmembrane receptor Patched 1. In doing so, it unleashes Smoothened from its inhibition, which, once released, transduces the Shh signal resulting in the activation of the zinc-finger transcription factors of the Gli family (81).

Shh-induced guidance of commissural axons is a complex process, involving cross-talk with the Wnt signaling pathways. Research findings have indicated that Shh has a function in attracting commissural axons toward the ventral midline during spinal cord development. Notably, Shh exhibits a dual effect on retinal ganglion cell axons: it attracts these axons at lower concentrations and repels them at higher concentrations (82). The importance of Shh in vascular regulation has been made clear across multiple studies which demonstrated its actions on arterial differentiation, vessel branching, and mural recruitment (83). Shh was shown to be a target of PDGF-BB signaling smooth muscle cells, where it is involved in cell migration, and its inhibition *in vivo* reduced the recruitment of NG2-positive cells to neovessels (83). In one such study, Shh-null mutants displayed an absence of vascular branching in the lungs during development (84). Shh was also shown to indirectly modulate angiogenesis through stromal cells in the perivascular niche, prompting them to express and release pro-angiogenic factors. These factors then operate within the vasculature, instigating the creation of new blood vessels that aid in enhancing the healing processes (85). Noncanonical Shh signaling, which operates independently of the transcriptional changes mediated by Gli transcription factors has also been shown to promote the formation of blood vessels through its effects on the cytoskeleton of endothelial cells by regulating PI3-kinase (86), as well as the small monomeric GTPases RhoA and Rac1 (87). These studies highlight the crucial role that the Shh pathway plays in angiogenesis.

*Netrins:* Netrins are proteins that guide migrating cells and axonal growth cones (88). Netrins have been found to exert their guidance activities via their action on the DCC (Deleted in Colorectal cancer) and Unc5 receptors (89). Netrins are involved in midline-crossing of commissural axons of the spinal cord through DCC. Netrin-1 has been shown to act both as a chemoattractant for ventrally directed commissural axons, and as chemorepellent for trochlear motor neurons (90, 91). In the PNS, Netrins have been demonstrated to play a role in guiding motor neurons to their target muscles. This was evidenced when mice lacking Netrin exhibited abnormalities in the projection of motor axons (92).

In blood vessels, inhibition of Netrin-1 binding to its receptor DCC suggested that Netrin-1 produced by arterial smooth muscle cells attract DCC-positive sympathetic axons toward the arterial wall (93). A 50% reduction of the expression of expression of Ntn-1 was sufficient to severely impair arterial innervation in small diameter resistance arteries branching off the iliac and femoral artery as well as arteries in the skin and internal organs, such as the esophagus and mesentery. On the other hand, overexpression of netrin-1 in the peritoneal cavity has also been shown to promote the ectopic extension of sympathetic axons away from the arteries, and this process could be blocked by blocking the netrin-1 receptor DCC (93).

Among the receptors for Netrins, the Unc5b receptor has been found to be selectively expressed in arteries and developing capillaries (94). This receptor, which has been reported to have a repulsive effect on endothelial cells, plays a role in controlling the morphogenesis of the vascular system, contributing to both normal developmental angiogenesis and pathological angiogenesis. A recent study also found that it regulates the integrity of the blood brain barrier by modulating Wnt/β-catenin signaling in brain endothelial cells (95). While some reports demonstrate that Netrin-1 negatively regulates capillary branching in the developing vascular system and to reduce endothelial cell migration *in vitro (* 94, 96), other researchers highlight its ability to positively modulate angiogenesis (97, 98). Netrin-1 was shown to promote NO production which then mediates Netrin-1-induced enhancement in endothelial cell growth and migration (99). Netrin-1 also promotes the survival of endothelial cells upon binding to UNC5B, which has been reported to promote apoptosis in the absence of ligand stimulation (100). The multifaceted role of Netrins in angiogenesis may be dependent on their localization as well as the expression of their receptors.

## 4 The nervous tissue within the tumor microenvironment

Several cellular and soluble components within the microenvironment influence tumor progression by contributing to the formation of new blood and lymphatic vessels (101). Similar to the processes of angiogenesis and lymphangiogenesis, evidence point out to the neoformation of nerve endings inside the tumors, termed neoneurogenesis (102). Nerve endings within tumors have been reported in several cancers such as skin, eye, heart, bladder, prostate, breast, pancreatic, esophageal, melanoma and colon cancers (103–109). Nerves fibers can facilitate tumorigenesis trough their effects on cancer cells directly or by modulating the microenvironment of tumors. It has also been suggested that the nervous system is functionally involved via the modulation of mediators of tumor progression (102). Increased neurogenesis has been associated with poor patient outcome, enhanced metastatic burden, and higher tumor grade in several models, including colorectal cancer (110), breast cancer (111, 112), pancreatic ductal adenocarcinoma (113), and thyroid cancer (114).The cross-talk between cancer cells and nerve fibers illustrates a synergistic epithelial-neural interaction in which both components secrete factors that favor their rapid growth. In this section, we describe several cellular and molecular processes by which neuronal cells can impact tumor progression and angiogenesis.

### 4.1 Perineural invasion (PNI)

Nerve fibers in tumors have been described to fulfill a structural purpose, acting as paths that enable the migration of perineural invading cells. This neural remodeling, which involves structural and functional changes in surrounding nerves within the tumor microenvironment results in increased nerve density, abnormal nerve branching, and altered nerve function, which in turn can create a supportive environment for tumor growth and can create a permissive environment for tumor cells to invade nerves and travel to distant sites, contributing to metastasis. Thus, it is likely that the perineural space is enriched in molecules that attract cancer cells and initiate a process called perineural invasion, which was defined as a coverage of at least 33% of the circumference of a nerve by a tumor (115). Research showed that pancreatic and prostate cancer cells are more proliferative and less susceptible to apoptosis when located near a nerve space (116, 117). The underlying mechanism could be the neoformation of axons via PNI based on the evidence that cancer cells secrete neurotrophic factors like NGF and axon guidance molecules like netrin-1 (118, 119). The incidence of PNI thus correlates positively with a poorer clinical outcome. In rectal cancer, the extent of PNI is even used as a prognostic factor (120).

*Neoneurogenesis*: Besides the ability of cancer cells to invade peripheral nerves, it has been proposed that cancer cells stimulate their own innervations. Therefore, the concept of neoneurogenesis also includes the development of nerve endings (axons) towards the tumor. This is supported by the fact that the shaping of the nervous system depends on a mechanism of extracellular matrix components assisted chemotactic axon guidance (121–123). A co-culture system with DRGs from the lumbar spinal cord of mouse embryos at 17 weeks of gestation and cancer cells was successfully used by Ayala and colleagues to 1) estimate neurite (or any projection from the cell body of a neuron) outgrowth, 2) neurite directionality towards cancer cells, 3) the existence of nerve-cancer cell interaction, 4) migration of cancer cells towards and through the nerves (PNI) and 5) the growth rate of cancer cells. They demonstrated that neurites extended from the DRGs to tumors *in vitro* in human pancreatic and prostate cancer (116, 117, 124). Although the stimulation of neoneurogenesis by tumor-released soluble factors has not yet been demonstrated in human cancers, the mechanism employed by cancer cells to induce neurite outgrowth could probably be similar to the process of nerve recovery after injury: unlike the CNS, the PNS can support long-axon regeneration after injury. In the adult PNS, Schwann cells or axon insulating cells, secrete growth factors that enable axonal regeneration, such as CNTF (ciliary neurotrophic factor), NGF, BDGF (brain-derived growth factor) and IGF (insulin-like growth factor) (125). Moreover, neurons require the activation of different signaling pathways by cancer cells to promote axonogenesis: GSK3 for microtubule stability and remodeling, PI3K for cell polarity, motility and chemotaxis and Akt for neuronal polarity (126). It has been revealed that growth factors and soluble factors that activate the aforementioned pathways, secreted by Schwann cells during axon regeneration, may also be secreted by cancer cells. Consequently, it is speculated that within the tumor microenvironment, axonogenesis could be under the control of cancer-derived signaling pathways similar to injury-induced axonal regeneration. Furthermore, cancer cells can generate their own extracellular matrix that favors axonal growth (124).

### 4.2 Neuroinflammation

Tumors can also trigger an inflammatory response in nerves, leading to the release of inflammatory mediators by the latter. These mediators, which include neuropeptides such as substance P and calcitonin gene-related peptide (CGRP), can promote tumor angiogenesis and increase the permeability of blood vessels, facilitating tumor growth and metastasis (127). The release of these neuropeptides and other inflammatory mediators can recruit also immune cells, such as macrophages and neutrophils, to the tumor microenvironment, which, in turn, release additional pro-inflammatory factors, growth factors, and cytokines that can stimulate tumor cell proliferation and survival. Neuropeptides released during neurogenic inflammation can also influence immune responses within the tumor microenvironment. Substance P and CGRP have been shown to enhance the recruitment and activation of immune cells, including T lymphocytes and dendritic cells (128). This modulation of immune responses can have both pro- and anti-tumorigenic effects, depending on the specific context.

Neurogenic inflammation mediators can also influence pain perception in cancer patients, as substance P and CGRP, released during neurogenic inflammation, are known modulators of pain sensations (128, 129). Additionally, neurogenic inflammation can impact the response to cancer treatments, such as radiation therapy or chemotherapy, by affecting the sensitivity of tumor cells to these therapies (130).

### 4.3 Neuronal-derived factors and tumor angiogenesis

Within tumors, nerve cells release several neurotrophic factors that can modulate the proliferation and phenotype of cancer cells, including Nerve Growth Factor (NGF), Neurotrophins (NT), Brain-Derived Neurotrophic Factor (BDNF) or Glial Cell Line-Derived Neurotrophic Factor (GDNF). In addition, axon guidance molecules and their receptors are also known to be related with tumor progression (102). Moreover, the literature shows that several neurotransmitters (epinephrine, norepinephrine, dopamine, serotonine, acetylcholine, glutamate, gamma-aminobutyric acid) and neuropeptides (neuropeptide Y, neurohypophyseal, corticotropin-releasing factor, opioid, secretin, somatostatin, tachykinin) are similarly involved in tumor progression, mainly by stimulating the proliferation or migration ability of cancer cells, a key event in metastasis (131). Although by themselves, these factors may not be critical for cancer cell survival, they were shown to facilitate tumor growth in the context of chemotherapy. Given that neurotransmitters and neuropeptides can activate signaling pathways pertaining to cell proliferation and survival, such as the PI3K, MAPK and Akt pathways, their inhibition should constitute a relevant chemo- or targeted therapy sensitization strategy (132). **Table 1** lists some of the reported effect of factors released by nerves within tumors and their reported effects on tumorigenesis. The effects of neuronal-derived factors and axon guidance molecules on the tumor microenvironment and angiogenesis are further discussed in the following sub-sections.

**Table 1:** Neurotrophic factors and their effects on tumor progression.

Neurotrophic Factor	Reported Effect	References
Nerve Growth Factor (NGF)	- NGF and its receptor TrkA are overexpressed in various types of cancer, including breast, ovarian, and prostate cancer. - Elevated NGF levels are associated with increased tumor growth, angiogenesis, and metastasis.	(133–140)
Brain-Derived Neurotrophic Factor (BDNF)	- Involved in the progression of several cancers, such as breast, colorectal, and lung cancer. - Increased BDNF expression has been associated with tumor growth, invasion, and resistance to chemotherapy.	(141–145)
Glial Cell Line-Derived Neurotrophic Factor (GDNF)	- Implicated in tumorigenesis; promotes the growth and metastasis of various cancers, including neuroblastoma and pancreatic cancer. - GDNF can stimulate angiogenesis and enhance the invasive properties of cancer cells.	(146–149)
Netrins/Unc5/DCC	- Netrin-1 and Netrin-4 delay the growth of lung, pancreatic and prostate tumors. - Netrin-1 promotes the growth of glioma cells by activating NF-κB signaling via UNC5A. - Netrin-1 is a positive regulator of malignant tumor metastasis by activating YAP signaling. - Netrin-1 induces antiapoptotic effects of acute myeloid leukemia cells through Unc5B. - Loss of netrin-1 receptor DCC is associated with a poor prognosis in patients with colorectal tumors, glioblastoma, and breast carcinoma. - Netrin-1 stimulates tumor progression in breast, cancer and medulloblastoma	(96, 97, 150–155)
Semaphorins/Neuropilins/Plexins	- High expression of Sema3F correlates with poor prognosis and tumor immune infiltration of hepatocellular carcinoma. - In breast cancer, Sema3A is a tumor suppressor, downregulated in tumor and negatively correlated with tumor stage. - In Lewis lung cancer, Sema3A binding to Nrp1 and PlexinA1/PlexinA4 coreceptors promotes tumor growth. - Sema4C is overexpressed in multiple types of malignant tumors, including breast cancer, esophageal cancer, gastric cancer, and rectal cancer. - Sema7A is involved in macrophage-mediated lymphangiogenesis in breast cancer. - Sema3A and Sema3B contribute to immune escape from the anti-tumor effects of CD8+ T cells.	(156–161)
Slit/ROBO	- Robo1 and Robo4 are significantly upregulated in colorectal carcinoma. - Slit2 and Robo1 are associated with an increased metastatic risk and decreased survival in colorectal carcinoma patients. - Increased Slit1 expression in prostate tumors - Slit/Robo signaling pathway plays a role in enhancing tumor cell migration and promotes tumor metastasis. - Slit2/Robo1 signaling upregulates MMP-9 to enhance breast cancer cell invasion. - Slit/Robo signaling decreases the proliferative rate and increases the apoptotic rate of oral squamous cells	(162–167)

### 4.4 Growth factors

Neurotrophic factors such as NGF and BDNF are known to play roles in tumor angiogenesis in various cancers, including breast cancer and chondrosarcoma. NGF regulates growth, differentiation and survival of peripheral neurons during embryonic development (168). In addition to its well-described action on survival and differentiation of neurons, NGF also acts as a pleiotropic molecule, involved in a wide variety of functions, such as neuropeptide modulation, wound healing and tissue scar. Endothelial cells have been shown to express the NGF receptors TrkA and p75 (169). Stimulation by NGF activates the Ras/ERK and PI3K/Akt signaling cascades in endothelial cells, both of which are involved in controlling cell proliferation and survival and are required for induction of endothelial cell migration (170).

NGF is upregulated in the tumor microenvironment of breast cancer. MDA-MB-231 breast cancer cells secrete NGF, which has been shown to stimulate angiogenesis *in vivo* when injected into immunodeficient mice (171). Moreover, NGF promotes the secretion of VEGF by breast cancer cells, and the angiogenic capacity of NGF is inhibited by *in vivo* administration of anti-VEGF antibodies. In human glioma microvascular endothelial cells, NGF plays a role in tumor angiogenesis by interacting with the α9β1 integrin (172).

Conversely, it was also shown that NGF can also inhibit the growth of HT1080 fibrosarcomas or HepG2 tumors via its effects on innervation and maturation of tumor neovasculature, which regulates blood flow into tumor tissues. Indeed, NGF markedly increased the density of α-smooth muscle actin (α-SMA)-positive cells in the vasculature of HT1080 tumors and significantly reduced blood flow in HepG2 tumors (173). A tumor-suppressive effect of NGF was also observed in prostate tumors by facilitating maturation of tumor blood vessels by migrating smooth muscle cells, and regulating tumor tissue blood flow (174).

BDNF is a neurotrophin essential for neuronal development and survival, synaptic plasticity, and cognitive function (175). BDNF acts on specific subtypes of TrkB-expressing neurons in the CNS and the periphery, and support the survival of existing neurons, as well as the growth and differentiation of new neurons and synapses. Studies have also shown that BDNF promotes angiogenesis by activating TrkB-expressing endothelial cells. The pro-angiogenic effects of BDNF were shown to be mediated via the PI3-kinase-Akt pathway, and modulation of BDNF levels directly correlate with Akt phosphorylation and inhibitors of PI3-kinase abrogate the BDNF responses in endothelial cells (176). BDNF can also indirectly induce angiogenic responses by regulating the expression of VEGF during tissue healing (177). BDNF was also shown to promote angiogenesis through the recruitment of Sca-1+CD11b+ pro-angiogenic hematopoietic cells which can facilitate neovascularization (178).

BDNF has been shown to contribute to tumor angiogenesis. In chondrosarcoma patients, the expression of BDNF and VEGF proteins is significantly higher and correlated with tumor stage. Knockdown of BDNF leads to decreased VEGF expression and abolishes angiogenesis in *in vitro* studies and animal models of chondrosarcoma (179). Co-transplantation experiments of BDNF-expressing endothelial cells with transformed mouse liver cells showed that high BDNF-expressing endothelial cells could facilitate tumor angiogenesis and growth of hepatocellular carcinomas whereas knockdown of BDNF by short hairpin RNAs impaired such effects (141). Immunohistochemical staining of human hepatocellular carcinomas revealed upregulation of BDNF and TrkB protein levels in both tumor and endothelial cells. High TrkB expression was associated with shorter overall survival. Interestingly, BDNF was also shown to regulate tumor lymphangiogenesis by modulating the expression of VEGF-C in human chondrosarcoma tissues (180). A positive correlation between TrkB expression and lymph vessel density was also observed in ovarian cancer (181) and is associated with the expression of VEGF-C and VEGF-D in oral squamous cell carcinoma (182).

### 4.5 Axon guidance molecules

Axon guidance molecules, including Semaphorins, Ephrins, Slits and their respective receptors Neuropilins, Eph and Robo receptors, have emerged as significant regulators of tumor angiogenesis. These molecules, which regulate axonal pathfinding during neural development, exhibit parallels in their ability to influence blood vessel growth and alignment within tumors (18).

Semaphorins and their receptors, often overexpressed in cancer cells, have been implicated in promoting angiogenic sprouting and guiding blood vessel trajectories. Neuropilin-1, a co-receptor for both Semaphorins and VEGF, stands out as a mediator of angiogenic signaling pathways. Several studies have shown a positive correlation between neuropilin-1 expression and exacerbated angiogenesis and poor prognosis in prostate, colorectal, kidney, lung and breast cancers (183). Neuropilin-1 expression in tumor endothelial cells, through its interaction with VEGF stimulates angiogenesis by enhancing VEGF signaling. It was shown that antibodies blocking VEGF binding to neuropilin-1 enhanced the anti-tumor effect of anti-VEGF antibodies (184). EG00229, an inhibitor of neuropilin-1 has also been shown to possess significant tumor-suppressive effects in gliomas and squamous cell carcinomas (185, 186). Furthermore, Sema3A selective mutants, which can competitively interact with PlexinA4 and prevent neuropilin-1/PlexinA4 interactions have also been shown to accelerate vascular normalization and reduce tissue hypoxia, increase perfusion and inhibit tumor growth in a mouse model of pancreatic cancer (187). Interestingly, in non-small cell lung cancer (NSCLC), there was an observed correlation between Sema4D expression and the development of perfused channels by tumor cells (188). This process, known as vascular mimicry (VM), allows tumors to create matrix-embedded vascular structures containing plasma and blood cells to fulfill the nutrient and metabolic requirements of neoplastic tissues (189, 190). Notably, this occurs without the involvement of pre-existing host endothelial cells. The resulting vascular-like structures do not constitute true blood vessels but rather imitate their function. Xia and colleagues demonstrated that heightened Sema4D expression was associated with an increased presence of VM channels in NSCLC tumors (188). The impact of Sema4D on VM was mediated through the RhoA/ROCK pathway, which governs tumor cell plasticity and migration. Consequently, inhibiting the expression of plexinB1 through RNAi-expressing vectors or blocking the RhoA/ROCK signaling pathway proved effective in reducing VM formation by NSCLC tumor cells (188).

Ephrins and Eph receptors, known for their role in repulsive axonal guidance, have been found to regulate endothelial cell migration and vessel alignment in tumor vasculature. The dual nature of Ephrin-Eph interactions, capable of either promoting or inhibiting angiogenesis depending on context, underscores their complexity in tumor microenvironments. Hypoxia and VEGF have been shown to cause the upregulation of ephrin-A1 in endothelial cells (191). Ephrin-B2 was also found to be uprregulated by VEGF and hypoxia. This was accompanied by increased tumor angiogenesis and stimulation of tumor growth (192). EphA2 forward signaling can also stimulate angiogenesis and it is known as a poor prognostic factor in endometrial cancer (193). Additionally, expression of Eph and ephrins has also been observed within lymphatic vessels, suggesting that it could regulate lymphangiogenesis (194). Therapeutically, it was shown that inhibition of Eph signaling using EphA2 and EphA3 Fc-fusion proteins could inhibit cancer progression and neoangiogenesis (195). Ephrin B1-derived peptides were also shown to suppress gastric cancer dissemination (196).

Netrins have also been implicated in tumor angiogenesis. It was shown that Netrin-1 binding to the Unc5B receptor on tumor endothelial cells limited tumor angiogenesis in models of lung, prostate and pancreatic cancer (96). Netrin-4, which was found to be overexpressed in VEGF-stimulated endothelial cells also negatively regulated angiogenesis by binding to neogenin, leading to the recruitment of Unc5B in PC3 xenograft tumors (150). Netrin-4 was also shown to delay colorectal cancer progression by inhibiting tumor angiogenesis (153). Conversely, netrins were also shown to possess pro-angiogenic activity in cancers. In tumors associated with the CNS, such as retinoblastoma and glioblastoma, netrin-1 did promote neovascularization (197). In patients with medulloblastoma, Netrin-1 also stimulated cell invasiveness and angiogenesis (198).

### 4.6 Neurotransmitter release

**Figure 2:**
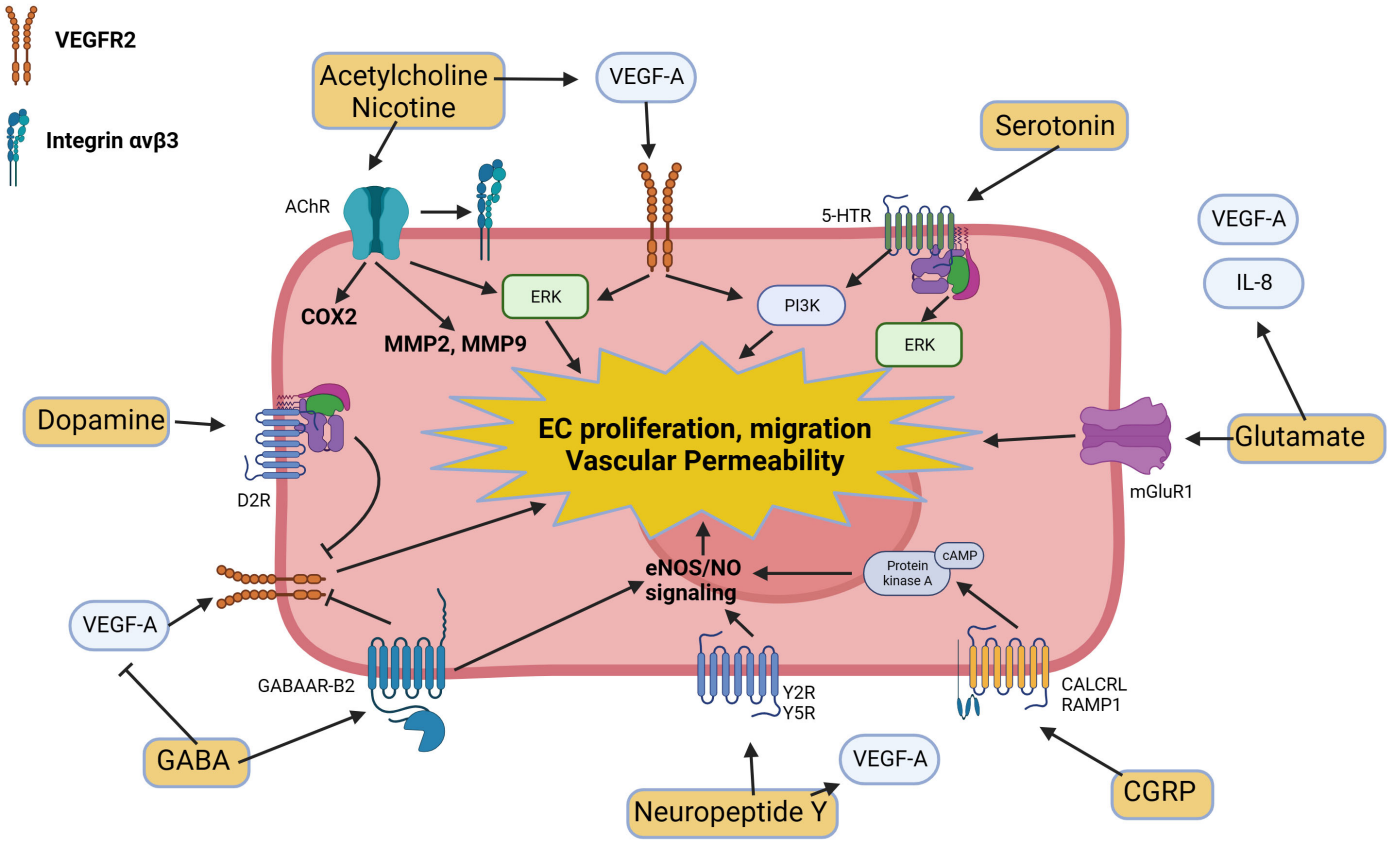
Molecular pathways triggered by neurotransmitters and neuropeptides in endothelial cells and their modulatory effects on angiogenesis.

Neurotransmitters in the tumor microenvironment exert significant effects on angiogenesis. These neurotransmitters and stress hormones can either promote or limit neovascularization through several mechanisms such as the regulation of expression of various angiogenic factors such as VEGF or metalloproteases (MMPs) or by modulating VEGF-induced endothelial cell proliferation, migration, and vascular permeability (**Figure 2**). The intricate interplay between neurotransmitters and angiogenesis suggests potential therapeutic opportunities for targeting the neuronal regulation of blood vessel growth in cancer treatment.

### 4.7 Dopamine

Dopamine exhibits antiangiogenic properties by blocking VEGF-induced endothelial cell proliferation, migration, and vascular permeability. The antiangiogenic properties of dopamine are mediated by the D2 dopamine receptors (D2R), leading to VEGF receptor 2 endocytosis, thereby preventing VEGF binding, receptor phosphorylation and subsequent signaling steps (199, 200). Studies have shown that dopamine significantly limits tumor neovascularization in several animal models of stomach, breast, and colon cancers (201–203). Depletion of peripheral dopaminergic nerves in mouse tumor models increased neovascularization and endothelial permeability (204), while treatments with exogenous dopamine inhibited tumor growth and angiogenesis (201). D2R agonists have also been found to abrogate lung tumor progression in syngeneic (Lewis Lung Carcinoma; LLC) and human xenograft (A549) orthotopic murine models and reduce tumor-infiltrating myeloid-derived suppressor cells (205). D2R knockout mice also display increased tumor growth due to exacerbated neovascularization (204). A positive correlation between endothelial D2R expression and tumor stage was also detected in lung cancer biopsies (205).

The dopaminergic system also plays a role in preventing vasculogenesis. Depletion of dopamine in the bone marrow leads to increased mobilization of endothelial progenitor cells (EPC) to sarcoma 180 (S180) tumors (206). Conversely, exogenous dopamine decreased VEGF-induced EPC mobilization, partly by reducing MMP-9 expression in the bone marrow.

### 4.8 Acetylcholine and nicotine

Acetylcholine receptors (AChRs) are expressed in various tissues, including the CNS and PNS, muscle, and non-neural tissues. At the neuromuscular junction, they are the primary receptors responsible for motor nerve-muscle communication, controlling muscle contraction (207). The main AChRs expressed in non-neuronal tissues include homomeric α7 AChRs as well as several heteromeric AChRs composed of α3, α5, β2, and β4 subunits, including α3β4-containing AChRs. Endothelial cells have been shown to express all mammalian AChR subunits with the exception of α2, α 4, γ, and δ (208). AChR signaling, triggered by endogenous agonists like acetylcholine or exogenous chemicals like nicotine, plays a role in promoting tumor angiogenesis. Stimulation of α7 AChRs can activate integrin αvβ3 and intracellular ERK MAP kinase signaling, leading to endothelial cell proliferation (209). This stimulation also induces the expression of various angiogenic factors, such as VEGF, PDGF, FGF, or TGF-β. Nicotine has also been shown to upregulate MMP-2 and -9 in endothelial cells, facilitating angiogenic sprouting (210).

In experimental and animal models of cancer, nicotine promotes angiogenesis and accelerates tumor growth. For instance, in mice with colon cancer tumors (HT-29), nicotine leads to elevated VEGF expression and increased microvascular density (211). Additionally, nicotine induces angiogenesis in lung cancer, where its systemic administration to mice bearing LLC tumors enhances tumor growth by accelerating tumor angiogenesis (38). Nicotine also promotes angiogenesis in gastric tumors through the upregulation of COX2 and VEGFR2 (212). In addition, nicotine also accelerated the growth of syngenic colon CMT93 tumor cells by inducing angiogenesis through the recruitment of bone marrow derived EPCs within the tumor (213). Selective antagonists of α7 AChRs, such as α-bungarotoxin and methyllycaconitine,have also been shown to inhibit endothelial cell proliferation, thereby limiting the angiogenic response (209) (214),. Isopropyl methylphosphonofluoridate, an irreversible inhibitor of acetylcholinesterase, the enzyme which catalyzes the breakdown of acetylcholine, also increased the number of newly forming blood vessels in L-1 sarcoma. This effect was mediated by the regulation of lymphocyte-induced angiogenesis and the modulation of angiogenic and pro-inflammatory cytokines secretion (215).

### 4.9 Y-aminobutyric acid (GABA)

Endothelial cells express various subunits of GABA_A_ receptors, including α1, α2, α6, β1, β2, β3, γ1, γ2, and γ3 subunits (216), where they participate to cerebral angiogenesis and neurogenesis (36). GABA_A_ signaling induces an inward Cl− current, a transient increase of calcium in endothelial cells and lead to the recruitment of endothelial nitric oxide synthase (eNOS) to produce NO, contributing to endothelial cell proliferation and angiogenesis (217). However, GABA_A_ receptor over-expression has also been shown to inhibit VEGF-induced endothelial cell proliferation, migration, and tube formation, as well as VEGFR-2 phosphorylation *in vitro*, and to reduce the phosphorylation of downstream PI3K components, such as PDK1, Akt, mTOR, TSC-2, p70S6K, and 4E-BP1 by directly binding with VEGFR-2 (218).

GABA stimulation has been shown to decrease VEGF levels in serum and tumor xenografts in stress-exposed mice (Panc-1 and BXPC-3) (219). Furthermore, GABA_A_ receptor overexpression also down-regulated the levels of VEGF and HIF-1α protein expression in a carcinoma model system (218). Additionally, in a mouse model of cholangiocarcinoma, GABA inhibited VEGF-A/C expression, resulting in decreased cell proliferation and tumor mass (220). GABAARB2, a GABA_A_ receptor subunit, was detected in the blood vessels of normal thyroid and thyroid tumors but not in thyroid cancer cells. This suggests that GABA signaling could also directly contribute to angiogenesis in thyroid cancer (221).

### 4.10 Serotonin

Serotonin is produced in the CNS and can be stored in platelets and presynaptic neurons (222). Its receptors (5-HTRs) are located on the cell membrane and are present in various tissues, including the CNS, heart, gastrointestinal tract, blood vessels, and platelets. The thrombotic environment of tumors induces platelet aggregation, leading to serotonin release, hence facilitating tumor angiogenesis. Serotonin has been found to enhance the migration, permeability, and proliferation of HUVEC and human aortic endothelial cells (HAEC) *in vitro* (223–225). This effect is mediated through the 5-HTR1, 5-HTR2, or 5-HTR3 receptors (225, 226). Serotonin also promotes the expansion of CD34-positive EPCs *in vitro* (227). Mechanistically, serotonin has been shown to trigger multiple signaling pathways in endothelial cells, including ERK, P70S6K, Src, PI3K, Akt, mTOR, and p38 MAPK (224, 228, 229).

Serotonin has been implicated in tumor angiogenesis. Tryptophan hydroxylase-deficient mice (serotonin-depleted) exhibited decreased microvessel density in MC38 colon cancers, along with higher MMP-12 and angiostatin concentrations in tumors (230). However, VEGF and VEGFR2 levels were similar to those of wild-type (WT) mice. In contrast, in a model of serotonin transporter-deficient mice bearing murine lung carcinoma or melanoma, tumor microvessel density was not affected, suggesting that the effects might be context-dependent (231). In human cancers, the presence of serotonin-positive cells was associated with higher VEGF expression and increased microvessel density in prostate cancer patients (232, 233). Serotonin could also regulate the vascular tone of the arterioles feeding the tumor through various 5-HTRs expressed on vascular smooth muscle cells, thereby regulating blood flow within the tumor (234).

### 4.11 Glutamate

Glutamate serves as an excitatory neurotransmitter and regulates synaptic activity through its receptors, including metabotropic glutamate receptors (mGluRs). These receptors are primarily expressed in the CNS, where they play a role in mediating neuronal excitability and neurotransmitter release. Studies have also shown that mGluR1, a subtype of mGluRs, is expressed in several human endothelial cell lines. Activation of mGluR1 signaling in endothelial cells has been linked to endothelial cell growth and tube formation (235).

Various cancers, such as melanoma, breast cancer, and gliomas, have been found to express different subtypes of mGluRs, implicating them in disease progression. Inhibition of mGluR1-mediated glutamate release resulted in decreased microvessel density in murine breast tumors (4T1) (235). Additionally, using BAY36-7620, a specific inhibitor of mGluR1, led to inhibited tumor growth and prolonged survival in mice with A549 or H1299 non-small cell lung tumors (236). The treated tumors showed reduced protein expression of bcl-2, HIF-1α, and VEGF, suggesting potential therapeutic implications. Increased expression of mGluR1 in several human melanoma cell lines was also associated with elevated expression of IL-8 and VEGF (237). This effect was attributed to the activation of the AKT/mTOR/HIF1 signaling pathway. Riluzole, a glutamate release inhibitor, was also shown to reduce microvessel density in breast cancer xenografts, demonstrating the significant role played by mGluR1 in tumor growth (238).

### 4.12 Neuropeptides

Neurons can secrete neuropeptides, including substance P and CGRP, which have been shown to play a role in modulating angiogenesis. Substance P has been identified as a promoter of angiogenesis. It achieves this by increasing the expression of pro-angiogenic factors and stimulating the proliferation of endothelial cells (239). Antagonists of the substance P receptor, Neurokinin-1 (NK-1R) promote apoptosis in tumor cells in a concentration-dependent manner, block the migration of cancer cells, prevent metastasis, and inhibit angiogenesis (240).

Neuropeptide Y (NPY) is secreted by tumor cells and exerts its effects through various receptors, particularly the Y2 receptor (Y2R). NPY plays a significant role in cancer development by mediating both proliferation and angiogenesis (241). The main mechanism by which NPY induces angiogenesis involves its direct influence on endothelial cells. NPY stimulates EC proliferation and migration *in vivo*, and it promotes the formation of capillary tubes (242). Moreover, *in vivo* studies have shown that endogenous NPY facilitates the vascularization of ischemic tissues (243). These actions are dependent on the activation of eNOS and, to some extent, on the VEGF pathway. NPY’s angiogenic activities are primarily mediated through Y2R, as demonstrated by impaired angiogenesis in Y2R-deficient mice (Y2R −/−) (244). NPY has also been shown to enhance the expression and secretion of VEGF, contributing to angiogenesis and promoting breast cancer progression (245). The mechanism underlying this effect involves the activation of endothelial cells through eNOS and through the modulation of the VEGF signaling pathway. NPY treatment also caused a significant increase in VEGF expression in 4T1 breast cancer cells, in a Y5R-dependent manner (245).

Administration of a Y2R antagonist (BIIE 0246) decreased tumor angiogenesis and serum VEGF concentration in obese mice with B16F10 melanomas without altering serum VEGFR1 or NO concentrations (246). Treatment with Y2R antagonists also inhibited angiogenesis of HT29 tumors by regulating the activation of the ERK/MAPK signaling pathway in endothelial cells (247). Y5R inhibition also reduced VEGF expression in 4T1 tumor cells (245). It has also been shown that tumor hypoxia can upregulate the expression of Y2R and Y5R in Ewing sarcoma and endothelial cells, which sensitizes them to the proliferative effects of NPY (248). Treatment of both neuroblastoma and Ewing sarcoma xenografts with a Y2R antagonist also resulted in a significant decrease in tumor vascularization (249). However, a Y5R antagonist alone did not inhibit vascularization of neuroblastoma xenografts (250).

CGRP is a neuropeptide widely distributed in the nervous system that exhibits numerous biological activities (251). It is a member of the calcitonin family of peptides and is produced in both peripheral and central neurons. It acts as a potent vasodilator and can function in the transmission of nociception. The CGRP receptors, Calcitonin Receptor-like receptor (CALCRL) and Receptor Activity-Modifying Protein (RAMP1), are ubiquitously expressed, suggesting that the protein may modulate a variety of physiological functions in all major systems. In endothelial cells, CGRP promotes cell proliferation (252) and activates eNOS via the cAMP-PKA pathway (253). *In vivo*, CGRP increases angiogenesis during placental development, wound healing and tumorigenesis (127, 254, 255). RAMP1 overexpression led to enhanced endothelial cell migration and capillary tube formation (256). Wound healing and wound-induced angiogenesis were also significantly suppressed in RAMP1(-/-) mice, and was associated with reduced expression of VEGF (257).

Using CGRP-knockout mice, it was shown that CGRP facilitates tumor angiogenesis, as tumor growth and tumor-associated angiogenesis in CGRP-knockout mice implanted with LLC tumors were significantly reduced compared with those in wild-type mice (127). The peptide CGRP 8–37, a CGRP receptor antagonist, was also found to block tumor growth and tumor-associated angiogenesis in wild-type mice (127). The expression of VEGF was also lower in the tumor stroma of CGRP-knockout mice compared to WT mice. CGRP precursor expression in the dorsal root ganglion in lung carcinoma-bearing mice were increased compared to those in mice with no tumors. Denervation of sciatic nerves suppressed LLC growth at the sites of denervation compared, suggesting that local expression of CGRP by peripheral nerves could drive tumorigenesis (127).

### 4.13 Sympathetic nervous system activation

Activation of the sympathetic nervous system, which is responsible for the “fight-or-flight” response, can affect tumor angiogenesis. Sympathetic or adrenergic nerves are closely associated with blood vessels. The release of catecholamines such as noradrenaline by such nerves mediates blood vessel constriction through surrounding smooth muscle cells contraction (258). Adrenergic nerves have also been shown to potentiate tumor angiogenesis by promoting a putative ‘angiometabolic switch’ in endothelial cells (259). Indeed, it was shown that the infiltration of adrenergic nerves in the microenvironment of prostate tumors resulted in the liberation of noradrenaline by nerve endings that subsequently stimulated the expression of the the ADRβ2 receptor gene in endothelial cells. When noradrenaline binds to ADRβ2, a change in endothelial cell metabolism occurs concomitantly with the inhibition of oxidative phosphorylation. As endothelial cells rely on aerobic glycolysis for angiogenesis, ADRβ2 activation promotes tumor angiogenesis which fuels cancer progression. This could be the cause of the observation that beta blockers improve the survival of patients with cancer: beta blockers inhibit sympathetic nerves mediated ADRβ2 signaling in endothelial cells which would in turn inhibit tumor angiogenesis. Evidence pointing to this mechanism is found in pancreatic cancer, where the use of beta blockers was correlated with a significant improvement of the survival rate of patients undergoing surgery, compared to control groups (260). β-adrenergic signaling also facilitates angiogenesis through a metabolic switch to activate glycolysis in prostate cancer endothelial cells (259).

Stress responses, which can lead to the release of Norepinephrine (NE) and Epinephrine (EPI), also enhance tumor neovascularization in primary ovarian tumors by upregulating the expression of VEGF and MMPs, which could also underlie the effects of β-blockers, which have been found to reverse stress-enhanced angiogenesis (261). Furthermore, the activation of β-adrenergic in melanoma, colon cancer, ovarian cancer and multiple myeloma has also been shown to lead to the release of IL-6 or IL-8, in addition to VEGF, contributing to the angiogenic response (262). Additionally, β-adrenergic signaling induces VEGF expression in tumor-associated macrophages through cAMP-mediated pathways (263, 264). Mechanistically, NE-induced VEGF expression was shown to be cAMP mediated, which in turn led to the activation of protein kinase A (PKA). β-adrenergic signaling also modulates the expression of MMPs, which degrade extracellular matrix components involved in tumor cell invasion and angiogenesis. Adrenergic stimulation upregulates MMP-2 and MMP-9 through the extracellular signal-related kinase 1/2 pathway in glioblastoma cells (265).

### 4.14 Sensory nerve-mediated regulation

Sensory nerves have been shown to be present in a several cancers, including cervical cancer, melanoma, gastric cancer, pancreatic cancer as well as basal cell carcinoma, where they can influence tumor angiogenesis through various mechanisms. Sensory nerves that travel alongside blood vessels promote angiogenesis via substance P-mediated signaling during inflammation and through its promotion of VEGF and MMP-9 expression in cancer cells (240, 266, 267). Sensory neuropeptides, CGRP and substance P, are also involved in the transcriptional upregulation of several angiogenic markers (VEGF, angiopoietin-2, type 4 collagen, MMP-2) in ECs (268, 269). Co-cultures of endothelial cells with sensory neurons also increased the protein level and enzymatic activity of matrix metalloproteinases 2 and 9 (MMP-2/MMP-9) in ECs (269).

It was also shown that sensory neurons could prevent tumor angiogenesis and growth. A recent study showed that depletion of Nav1.8-positive sensory neurons using genetic or pharmacological approaches could enhance melanoma growth and tumor angiogenesis (103). In this study, Prazeres and colleagues demonstrated that ablation of nociceptors in a murine model of B16F10 melanoma enhanced tumor growth and the intra-tumoral blood vessels’ area, suggesting that sensory nerves inhibit tumor angiogenesis in this model. Furthermore, using silencing of genetically defined neuronal populations upon binding to small-molecule designer drugs, a 2021 study showed that inhibition of sensory neurons’ activity is sufficient to trigger an increase in melanoma growth and intra-tumoral new blood vessel formation (270). Conversely, chemogenetic stimulation of sensory neurons counteracted melanoma progression, by negatively regulating tumoral growth, angiogenesis and immunosurveillance (270). While the underlying mechanisms by which nociceptors could limit tumor angiogenesis in melanoma remain unclear, it has been speculated that neuropeptides such as substance P, VIP, tachykinins or CGRP could play a role. Furthermore, sensory nerves may tune down sympathetic nerve activity (271), limiting norepinephrine release, which has been shown to strongly induce tumorigenesis and angiogenesis (262, 272, 273).

### 4.15 Neurovascular coupling

Neurons and blood vessels in the brain are tightly interconnected, and neurovascular coupling refers to the coordination between neuronal activity and local blood flow regulation (29). Changes in neuronal activity can influence the supply of oxygen and nutrients to surrounding tissues, including tumors (274). Disruptions in neurovascular coupling, such as alterations in blood flow regulation, can affect tumor angiogenesis, and perturbations of the neurovascular unit composed of astrocytes, pericytes, and endothelial cells have been described in tumors of the nervous system such as gliomas (274–277).

Recent studies have highlighted the presence of neurovascular uncoupling in association with brain tumors. These disruptions interfere with neurovascular coupling, resulting in excitotoxicity caused by an inadequate vascular response, leading to decreased delivery of oxygen and nutrients, resulting in hypoxia and cell death, initiating a chain of events that ultimately triggers angiogenic growth and the development of poorly formed vasculature (278, 279). Furthermore, it has been suggested that the failure to deliver essential metabolites required for neural firing may accelerate excitotoxicity (279).

Glioma cells have been shown to closely interact with the brain vasculature, migrating along blood vessels and using them as conduits during tumor spread, leading to disruption of structural and functional integrity (279, 280). These interactions further suggest that tumors could impact the coupling between neuronal activity and vascular dynamics during glioma-infiltration and contribute to the disturbed functional and metabolic state of the brain during tumor progression. Indeed, a recent study demonstrated that glioma infiltration led to tumor-localized alterations in neuronal synchrony and neurovascular coupling, interictal events, and seizures accompanied by disrupted hemodynamic responses in mice (274). Changes in vascular perfusion, combined with the marked increase in neuronal activity and metabolic demands, were postulated to lead to tissue hypoxia within the tumor-bearing cortex, which in turn would drive the expression of cytokines and growth factors that stimulate microvascular proliferation.

Apart from neurovascular uncoupling, the growth of tumor cells along the abluminal surface of cerebrovasculature and the disruption of astrocyte function appear to compromise the integrity of the blood-brain barrier tight junctions in gliomas. As a result of gliomas down-regulating tight junction proteins (claudin-1, -3, and -5), the blood-brain barrier becomes more permeable (278, 281). This phenomenon leads to the formation of leaky blood vessels which can accelerate pro-inflammatory responses. Moreover, glioma growth on the abluminal surface of blood vessels can also impair the association of pericytes with the vasculature, contributing to blood-brain barrier breakdown. Consequently, a decrease in pericyte population may not only affect blood-brain barrier permeability, allowing the entry of molecules that are normally filtered out into the brain’s extracellular matrix, but also result in neurovascular uncoupling (278).

## 5 Neurogenesis and tumor progression

A recent study on prostate cancer used lineage tracing of doublecortin-positive neural progenitor cells from the subventricular zone of the brain to examine whether tumor innervation can originate from the CNS (282). It was observed that subventricular zone-derived neural progenitor cells could cross the blood-brain barrier and migrate to the site of the tumor to further populate it. Upon arrival in the tumor microenvironment, these neural progenitor cells mature to form sympathetic nerves that promote tumorigenesis (283). Depletion of these doublecortin-positive neural progenitor cells abolishes tumor initiation and progression. Conversely, transplantation of doublecortin-positive cells from the subventricular zone augments tumor xenograft growth and metastasis *in vivo* (282). Furthermore, lineage tracing of ex vivo-tagged human gastric and colorectal carcinoma stem cells, which are cells that can both self-renew and differentiate, showed that these cells not only initiated tumorigenesis in xenograft mouse models but also that the tagged human cancer stem cells differentiated into tyrosine hydroxylase-producing sympathetic and vesicular acetylcholine transporter-producing parasympathetic neurons upon engraftment. Inhibition of their differentiation capacity abrogated xenograft tumor growth (284). These data support the notion that cancer stem cells can mature into heterogenous cell populations, including neuron-like cells, that are found in the tumor microenvironment. Moreover, stem-like progenitors in prostate cancer possess neurogenic gene expression profiles (285).

## 6 Concluding remarks

This review evaluated the signaling pathways and molecules within the neural systems and examined their potential roles on the development of blood vessels within tumors. The identification of neurons-derived factors within the tumor microenvironment can shed light on molecular processes involved in tumorigenesis. Indeed, forthcoming studies have the potential to enhance existing anti-angiogenic or vascular normalization therapies by delving deeper into the molecular mechanisms underlying angiogenesis and neurogenesis. By studying the impacts of various combinations of neuron-derived factors and guidance cues on endothelial cells and analyzing the factors that modulate the balance between sprouting angiogenesis and quiescence in endothelial cells, we can significantly advance our comprehension of the intricate mechanisms involved in the establishment of the tumor microenvironment and design strategies to improve therapeutic strategies.

## Author contributions

SS: Writing – original draft. AB: Conceptualization, Funding acquisition, Supervision, Writing – review & editing. LB: Conceptualization, Funding acquisition, Supervision, Writing – original draft, Writing – review & editing.
